# Light Control-Induced Oxygen Vacancy Generation and In Situ Surface Heterojunction Reconstruction for Boosting CO_2_ Reduction

**DOI:** 10.3390/molecules28104057

**Published:** 2023-05-12

**Authors:** Zhimin Yuan, Xianglin Zhu, Qichao Gao, Zaiyong Jiang

**Affiliations:** 1 School of Chemistry & Chemical Engineering and Environmental Engineering, Weifang University, Weifang 261061, China; 2 Institute for Energy Research, School of Chemistry and Chemical Engineering, Jiangsu University, Zhenjiang 212013, China; 3 School of Light Industry and Engineering, South China University of Technology, Guangzhou 510640, China

**Keywords:** photocatalytic CO_2_ reduction, oxygen vacancy, surface heterojunction reconstruction, BiOBr photocatalyst, visible light utilization

## Abstract

The weak adsorption of CO_2_ and the fast recombination of photogenerated charges harshly restrain the photocatalytic CO_2_ reduction efficiency. The simultaneous catalyst design with strong CO_2_ capture ability and fast charge separation efficiency is challenging. Herein, taking advantage of the metastable characteristic of oxygen vacancy, amorphous defect Bi_2_O_2_CO_3_ (named BO_v_C) was built on the surface of defect-rich BiOBr (named BO_v_B) through an in situ surface reconstruction progress, in which the CO_3_^2−^ in solution reacted with the generated Bi^(3−x)+^ around the oxygen vacancies. The in situ formed BO_v_C is tightly in contact with the BO_v_B and can prevent the further destruction of the oxygen vacancy sites essential for CO_2_ adsorption and visible light utilization. Additionally, the superficial BO_v_C associated with the internal BO_v_B forms a typical heterojunction promoting the interface carriers’ separation. Finally, the in situ formation of BO_v_C boosted the BOvB and showed better activity in the photocatalytic reduction of CO_2_ into CO (three times compared to that of pristine BiOBr). This work provides a comprehensive solution for governing defects chemistry and heterojunction design, as well as gives an in-depth understanding of the function of vacancies in CO_2_ reduction.

## 1. Introduction

The over-reliance on fossil fuels has boosted the industrialization of the world during the past hundred years; however, it also caused the emission of a large amount of greenhouse gas carbon dioxide (CO_2_) [1,2]. Controlling or reducing the concentration of CO_2_ in the atmosphere is very important for addressing mentioned environmental problems [3]. Several prevalent strategies, such as electrochemical CO_2_ reduction [4], CO_2_ hydrogenation [5,6], and photocatalytic CO_2_ reduction [7,8], were developed as potential solutions for future CO_2_ capture and conversion. In 1978, Halmann et al. first reported the photocatalytic reduction of CO_2_ to produce chemical fuels by using a semiconductor photocatalyst. The photocatalytic CO_2_ reduction has attracted more and more attention. Up to now, many semiconductor-based materials, such as ZnO, GaN, ZrO_2_, Bi_2_WO_6_, TiO_2_, and C_3_N_4_, have been developed as functional catalysts for photocatalytic CO_2_ reduction, proving that CO_2_ can be converted into CO, CH_4_, methanol, and other valued chemicals using H_2_O sacrificial agent [9,10,11,12,13,14,15,16]. After decades of exploration and development, many excellent achievements have been obtained, but the practical application of photocatalytic CO_2_ reduction is severely limited by the low activity and the poor stability of the existing catalysts. Theoretically, the final efficiency of solar energy utilization is determined by three steps: light capture and carrier generating, migration and separation of electron-hole pairs, and surface reduction at the active sites. Accordingly, the ameliorated efficiency of the above-mentioned steps synergistically is the emphasis on photocatalyst design.

Bismuth oxyhalides BiOX (X = Cl, Br, and I), as sillén structure materials containing [Bi_2_O_2_] layer interleaved between two X layers, have attracted worldwide focus in the photocatalysis field recently given rise to prominent properties, including composition adjustability, chemical stability, low toxicity, and inexpensiveness [17,18,19,20]. Nevertheless, the photocatalytic performance of BiOX catalysts is still limited due to the fast recombination of carriers and the lacking catalytic active sites [21,22]. Currently, many approaches have been explored to improve the separation efficiency of photogenerated carriers of BiOBr catalysts, such as doping with other metal or nonmetallic atoms, surface vacancy designing, morphologies adjustment, heterojunction construction, and cocatalyst modification, etc. [23]. The internal mechanism for enhancing the photocatalytic activities in the above approaches can be typically explained in three facts: extending the light absorption, promoting the separation of the carriers, and building more active reaction sites, which are also considered as main challenges in highly efficient catalyst designing and future practical application. After the photocatalysts were excited by a certain wavelength of light, photo electron holes were produced. While the migration distance of the generated carriers is usually limited, and this means not all the carriers can migrate to the surface of catalysts. Most of the photo-generated electron-hole pairs are recombined during transmission. What is more, the further going on of the reaction needs appropriate reaction sites. The final finish of the photocatalytic reaction must combine all the above steps. In a word, photocatalytic reactions are complicated, and either of the steps can be the rate-determining step. The designing of highly active catalysts is a systematic project. Among the BiOX catalysts, BiOBr shows visible light response-ability and has a proper band gap position compared to the BiOCl and BiOI, which are good candidates for CO_2_ reduction. Recently, lots of effort have been performed to improve the activity of the BiOBr. Wu et al. [24] prepared a kind of Gd^3+^ doped BiOBr material, and they found that the doping of Gd^3+^ can widen visible light response compared to the pure BiOBr. Additionally, the Gd^3+^ doped BiOBr more negative conduction band position, which is beneficial to CO_2_ reduction. The enhanced light response was considered the main reason for the improved performance. Mi et al. designed a series of BiOBr nanosheets with exposed different sizes and crystal facets. [25] Due to the surface energy difference of different facets, an internal electric field is formed between the facets, which can force the migration and separation of the photo electron-hole pairs. As a result, the activity of the BiOBr nanosheet was improved. Constructing heterojunction is the widely used approach for facilitating interface carrier separation. Giving rise to the potential bandgap differences, the formed internal electric field at the surface can separate the carriers [23,26]. For instance Guo’s team reported a novel Bi/BiVO_4_/V_2_O_5_ and the properties of the ternary catalyst in water oxidation were studied. The optimized Bi/BiVO_4_/V_2_O_5_ exhibited a much better activity than BiVO_4_ catalyst. The authors prove that the enhanced performance was attributed to the synergistic effect of the formed Bi/BiVO_4_/V_2_O_5_ heterojunction structure, which can greatly enhance the separation efficiency of the photogenerated carriers [27]. In another recent research, a Z-scheme Bi_4_TaO_8_Cl/W_18_O_49_ heterostructure was constructed and used as a CO_2_ reduction photocatalyst. Utilizing the unique properties of Bi_4_TaO_8_Cl nanostructure and the merits of oxygen vacancy in W_18_O_49_, the carrier migration channels between the Bi_4_TaO_8_Cl and W_18_O_49_ were built along with Z-scheme to boost the separation of the photogenerated carriers. Recently, surface vacancies have attracted an amount of attention in the photocatalytic CO_2_ field, and it is proven that the existing defects in proper concentration can enlarge the CO_2_ reduction efficiency. For example, Xie et al., have reported the existence of oxygen vacancies in BiOBr could create an intermediate level, resulting in the extension of its light response [28]. What is more, the oxygen vacancies are helpful for the adsorption and activation of carbon dioxide and are proven to the forming of COOH* intermediate.

All the above strategies are ingenious in building transmission channels or creating activation sites; however, how to systematically integrate the above advantages used in different tactics is still a huge challenge and rarely reported. Recently, the importance of surface reconstruction theory was proposed and developed to design highly efficient catalysts. The surface reconstruction theory also helps to understand the true catalytic active site of catalysts. Kibria and the co-authors made use of the surface reconstruction route in the preparation of the CO_2_ electroreduction catalyst [29]. Using CuCl as the precursor, a Cu-based catalyst owing to the advantages of oxidation state and morphology was constructed through a wet-oxidation method, which helps the tuning of C_2+_ selectivity in CO_2_ reduction. Li’s group prepared an oxygen-doped BiSI catalyst containing rich sulfur vacancies utilizing the surface reconstruction route. The surface BiSI was oxidized slightly by controlling the reaction conditions, which caused the generation of an O-doped BiSI layer. As a result, a special BiSI/O-doped BiSI catalyst was constructed and showed an enhanced Cr(VI) reduction activity because of the formed tight contact interface, which can hugely boost the migration of the photogenerated carriers and help the adsorption of the Cr(VI) on the surface [30].

Here, in this work, based on the chemical nature of vacancies in BiOBr material and the surface reconstruction strategy, a novel BO_v_B/BO_v_C photocatalyst was prepared using BiOBr as raw material through an in-situ surface reconstruction induction progress. In detail, oxygen vacancies rich BiOBr was first prepared through a UV light irradiation method. Under the irradiation of UV light, the deep-level electrons were excited, and some of the Bi^3+^ atoms were reduced to a lower valance state which induced the formation of oxygen vacancies. During the photocatalytic CO_2_ reduction progress, the defect sites were attacked by CO_3_^2−^ in solution and generated amorphous BO_v_C, which has a mass of vacancies. This kind of formed heterojunction was caused by in situ phase-changing progress, which contains a tight interface and benefits the transferring of electrons. The amorphous BO_v_C contains amounts of oxygen vacancies that are pivotal for the adsorption and activation of carbon dioxide. This study offers a thorough understanding of how to design advanced photocatalysts with synergistic defect and heterojunction engineering advantages.

## 2. Results and Discussion

### 2.1. Structural Characterization and Morphological Analysis

**Figure 1:**
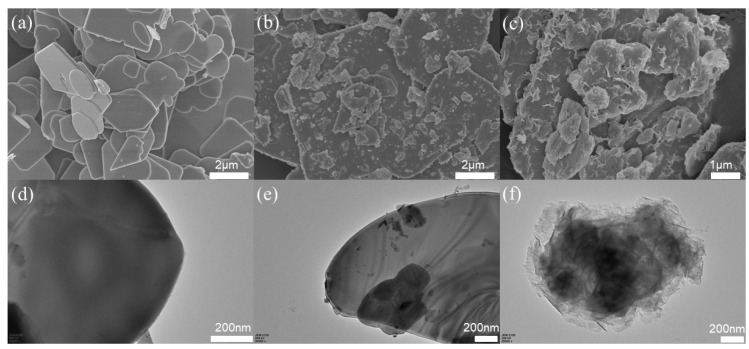
(**a**–**c**) SEM and (**d**–**f**) TEM images of the pristine BOB, BO_v_B-5, and BO_v_B/BO_v_C-5 photocatalysts.

The morphologies changing process were investigated with the Scanning electron microscope (SEM) and Transmission electron microscope (TEM). Figure 1a–c and d–f presents the SEM and TEM images of pristine BOB, BO_v_B, and BO_v_B/B_2_O_v_C-5 photocatalysts, respectively. From the SEM results, it is found that the pristine BOB sample is composed of micro sheets with smooth surfaces. After the irradiation treatment, much fragmentation occurred on the surface BO_v_B, which is due to the morphology structure destruction derived from the stirring process. Through the final reaction in saturated CO_2_ solution, nanoflakes formed on the surface of the micro sheets, and the surface transformation maybe is caused by the conversion of BOBr to Bi_2_OCO_3_. Similar results are also observed in the TEM images, and it can be concluded from Figure 1f that BO_v_B/BO_v_C are composed of a shaggy shell and crystalline core, which is entirely different from the pristine BOB (Figure 1d) and BO_v_B (Figure 1e). Additionally, the surface morphologies of all BO_v_B samples were presented in Figure S1, and it is clearly observed that there is much more fragmentation occurred on the surface BO_v_B with the prolonged irradiation time.

**Figure 2:**
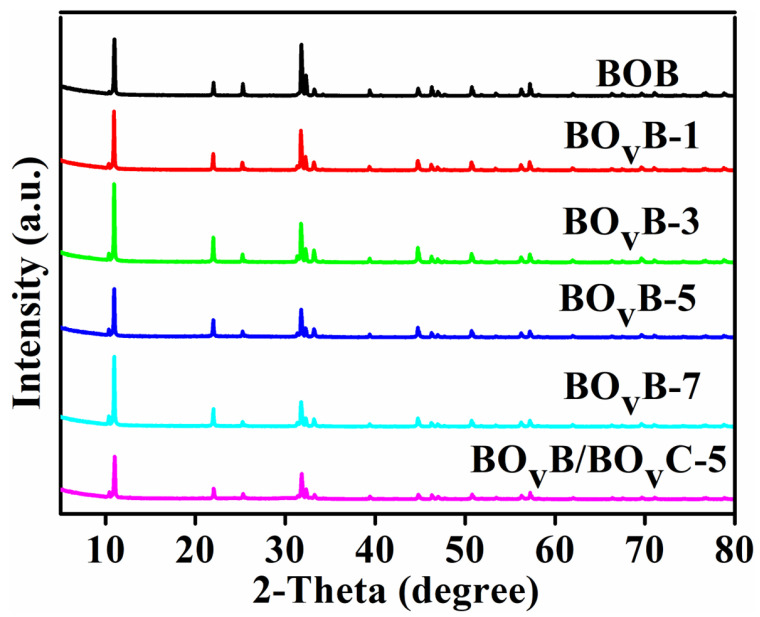
XRD patterns of pristine BOB, BO_v_B, and BO_v_B/BO_v_C-5 photocatalysts with tuning the irradiation time.

The crystal structure of the prepared BOB sample was characterized, and the results were presented in Figure 2. As shown in the XRD patterns, both the pristine BOB and UV light-treated samples have intense and distinct diffraction peaks, which indicate the purity and good crystallinity of the samples. It also means the forming of oxygen vacancies didn’t destroy the major structure of the BOB. The series of peaks at around 2θ degree of 10.9, 21.9, 25.2, 32.2, 39.4, and 46.2 correspond to the (0 0 1), (0 0 2), (1 0 1), (1 1 0), (1 1 2), and (2 0 0) planes, respectively, which response to the BOB (JCPDS No. 09-0393) [31]. In addition, the diffraction intensity of (1 1 0) gets weaker with the prolong of the irradiation time, which can be due to the replacement of the oxygen atoms by oxygen vacancies, which weakens crystallinity. In addition, the BO_v_B/BO_v_C-5 sample obtained after a photocatalytic reaction has been performed the XRD test. As shown in Figure 2, it should be noted that we did not find the diffraction peaks of Bi_2_OCO_3_ after CO_2_ reduction progress, and this can be due to the amorphous properties of the formed Bi_2_OCO_3_.

### 2.2. Analysis of UV-Vis Absorption Spectra

**Figure 3:**
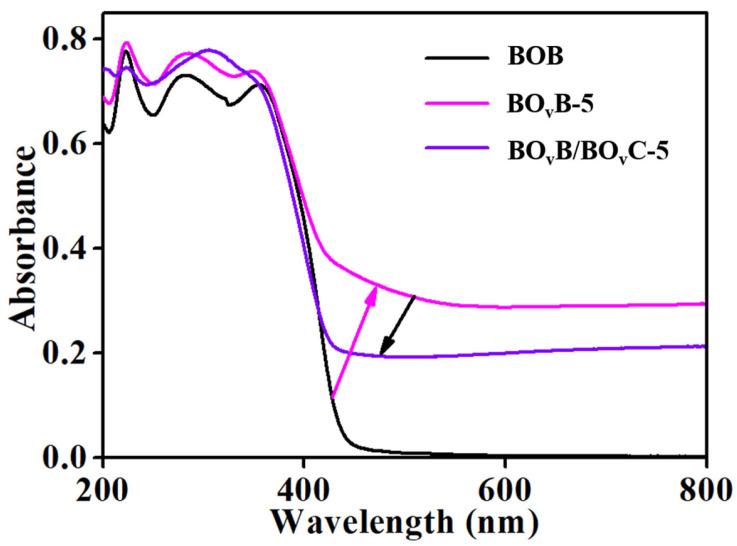
UV–vis DRS spectra of different photocatalysts.

As it is known that the forming of oxygen vacancies will induce the generation of defect states, the presence of defect states will fabricate an intermediate energy level near the conduction band [32]. Theoretically, the intermediate energy level can accept the electrons excited from the valance band, in turn causing the broadening of the light absorption range. To further illustrate the influence of oxygen vacancy defects for enhanced photocatalytic performance, we investigate the optical properties of pristine and vacancies-rich samples through UV/Vis diffuse reflectance spectra. As shown in Figure 3, both vacancies-rich BO_v_B-5 and BO_v_B/BO_v_C-5 present strong absorption in the range of the visible light region compared to the pure BOB. While the absorption intensity of BO_v_B/BO_v_C-5 gets weaker compared with the vacancies-rich BO_v_B-5. From the optical properties, we can conclude that the existence of oxygen vacancy does affect light absorption properties and widen the light response region. The weakened light absorption intensity of the BO_v_B/BiO_v_C-5 sample indicates the consumption of defects by the ${\mathrm{CO}}_{3}^{2-}$.

### 2.3. Raman and EPR Analyses

**Figure 4:**
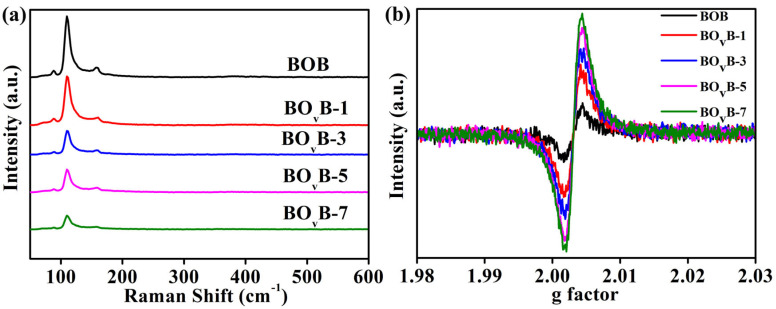
Raman (**a**) and EPR (**b**) analyses results of different photocatalysts.

The generation and vacancies concentration in the catalysts were further characterized using Raman spectroscopy and EPR spectra tests, as shown in Figure 4. In the Raman spectra results (Figure 4a), the peaks located at around 91 and 113 cm^−1^ are assigned to the signal of the A1g internal Bi-Br stretching mode, whereas the weak peak at 162 cm^−1^ is related to the Eg internal Bi-Br stretching mode [32,33]. It apparently regularly weakens the Raman peaks by prolonging the irradiation time, which can be attributed to the gradual distortion of the crystal structure after the inducing of oxygen vacancies. To further prove the relation between oxygen vacancies generation and the irradiation operation, electron paramagnetic resonance (EPR) analyses tests were given, as shown in Figure 4b, and the signals significantly enhanced at around *g* = 2.003 as the prolonging of the irradiation time, which means the increase of the vacancy’s concentration [33,34].

### 2.4. XPS Characterization

**Figure 5:**
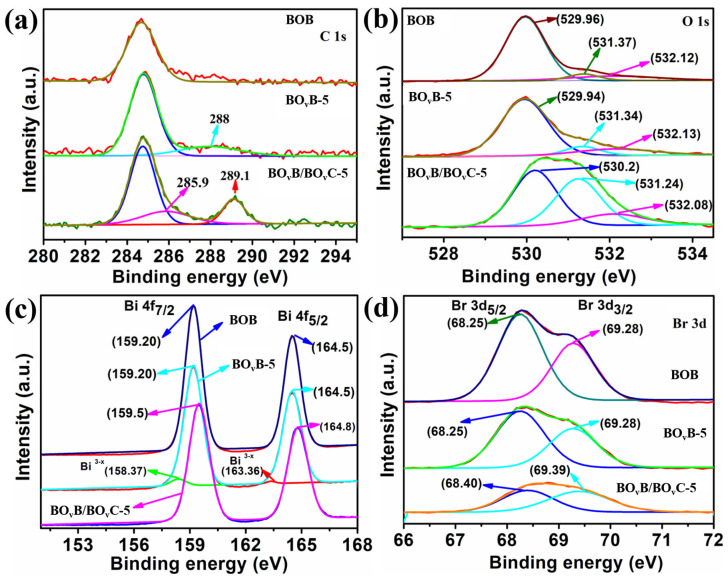
XPS spectra of as-prepared different photocatalysts, (**a**) C 1s, (**b**) O 1s, (**c**) Bi 4f and (**d**) Br 3d.

The surface chemical composition change progress during the reaction was further characterized through X-ray photoelectron spectroscopy (XPS) technology, and the spectrum results are presented in Figure 5. In the C1s spectrums (Figure 5a), the existing single peak at 284.6 eV excludes the influence of carbon impurity on the surface of the pristine BOB. After irradiation 5 h, there is one obviously raised peak at around 288 eV, and this peak is attributed to the surface absorbed CO_2_ [35,36,37]. As is known, the oxygen vacancies at the material surface are metastable and can be oxidized or occupied by other anions, and based on this rule; the vacancies-rich BO_v_B-5 was treated in the saturated CO_2_ solution. From the results, it can be seen that two peaks at 285.9 and 289.1 eV appeared, which responded to the binding energy of C–O and C=O groups of the ${\mathrm{CO}}_{3}^{2-}$ [31]. The insertion of ${\mathrm{CO}}_{3}^{2-}$ can also be confined in the O1s spectrums in Figure 5b, three similar peaks occurred at around 520, 531, and 532 eV in both BOB, BO_v_B-5, and BO_v_B/BiO_v_C-5 samples, which corresponded to the lattice oxygen, and oxygen vacancies, and surface adsorbed oxygen species, respectively [38]. It is worth noting that the peak intensity of BO_v_B and BO_v_B/BiO_v_C at 532.1 eV was much more enhanced than the BOB sample, indicating the higher intensity of oxygen vacancy. In addition, the BO_v_B/BiO_v_C-5 sample owned a stronger surface adsorbed oxygen peak, indicating the insertion of ${\mathrm{CO}}_{3}^{2-}$ [36,39]. In addition, the oxygen vacancy intensity of BO_v_B/BiO_v_C is also enhanced compared with BiO_v_C-5, which may be caused by the amorphous property of surface Bi_2_O_v_CO_3_. The low valance Bi_3−x_ signal peak in the Bi 4f spectrum of BOB-5 sample (Figure 5c) also illustrates the formation of oxygen vacancies. The binding energy around 68.2 and 69.3 eV is related to Br 3d5/2 and 3d3/2 respectively (Figure 5d), which is assigned to the monovalent oxidation state Br [40]. The XPS results elucidate the forming progress of oxygen vacancies and heterojunction structure.

### 2.5. Researches on Photocatalytic Performance and CO_2_ Reaction Path

**Figure 6:**
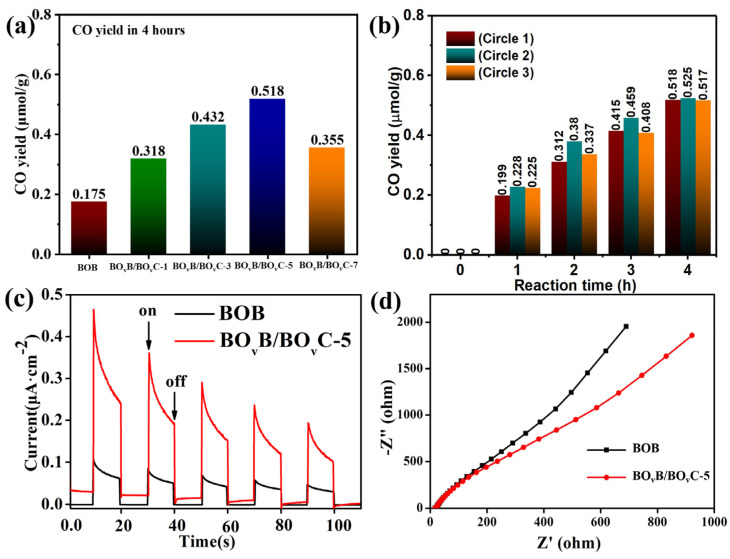
(**a**) Photocatalytic CO_2_ reduction experiment with different catalysts, (**b**) Photocatalytic CO_2_ reduction stability tests, (**c**) Transient photocurrent responses (**d**), and electrochemical impedance spectra of the samples under visible light irradiation (wavelength > 420 nm).

The photocatalytic CO_2_ reduction performance of the prepared catalysts was evaluated in a quartz reactor containing saturated CO_2_ under visible light irradiation (λ > 420 nm), and the temperature of the quartz reactor was steadily kept at 15 °C. Figure 6a is the results of CO yield in 4 h, and it was found that the activity was gradually enhanced with the increasing intensity of oxygen vacancies, and the BO_v_B/BO_v_C-5 shows the best CO_2_ reduction activity of 0.518 μmol/g, which is nearly 3 times of the pristine BOB (0.175 μmol/g). As mentioned, the formation of BO_v_C relied on the generation of oxygen vacancies, which can provide a mass of low-valance Bi^3−x^ to react with the $\;{\mathrm{CO}}_{3}^{2-}$ and form BO_v_C. The CO_2_ adsorption isotherms were performed under ambient conditions (298 K), and the results are shown in Figure S2. It could be observed that adsorption capacity is linearly related to the oxygen vacancy concentrations, which also illustrates the critical role of vacancies in the BO_v_B-X. The decay of activity was owing to formed recombination centers caused by the existence of excess oxygen vacancy. The enhanced activity indicates the success of the surface modification strategy. The stability of the photocatalyst was also investigated, and the results are presented in Figure 6b. In the three cycles test, the activities have no obvious change, proving the good stability of the catalyst. To investigate the internal mechanism of the CO_2_ reduction reaction, the transient photocurrent and electrochemical impedance spectra (EIS) tests were carried out to confirm the generation and separation properties of the carriers. As shown in Figure 6c, the BO_v_B/BO_v_C-5 exhibits a higher photocurrent response compared with the pristine BOB and oxygen defect BOB. The EIS results (Figure 6d) indicate the interfacial charge transfer efficiency and the smaller arc radius of the EIS Nyquist plots means smaller charge transfer resistance. As presented, BO_v_B/BO_v_C-5 shows the best separation efficiency of the carriers.

**Figure 7:**
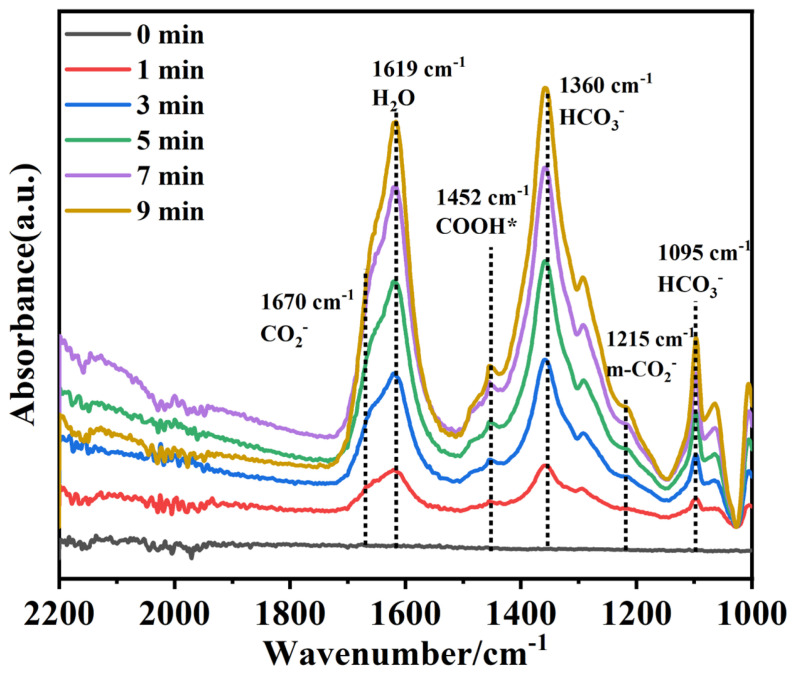
In situ FT-IR spectra of BO_v_B/BO_v_C-5 sample.

To deeply understand the possible paths of CO_2_ reduction, the in situ FTIR spectra were used for the signals collection of the reaction intermediates, as shown in Figure 7. As the reaction went on, the characteristic absorption peaks of HCO_3_^−^ (1095 cm^−1^ and 1360 cm^−1^), m-CO_2_^−^ (1215 cm^−1^), CO_2_^−^ (1670 cm^−1^), and COOH* (1452 cm^−1^) were clearly identified in the spectra results. From the in situ FTIR results, it can be concluded that the CO_2_ molecules were fixed onto the surface of the catalyst and formed into HCO_3_^−^. Then, the photogenerated electrons were captured by HCO_3_^−^ and CO_2_^−^ was produced. The generated m-CO_2_^−^ was further transferred to COOH*, which is the key intermediate for CO evolution. The in situ FTIR results can give clear proof for the CO evolution path [41,42].

### 2.6. Mechanism

**Figure 8:**
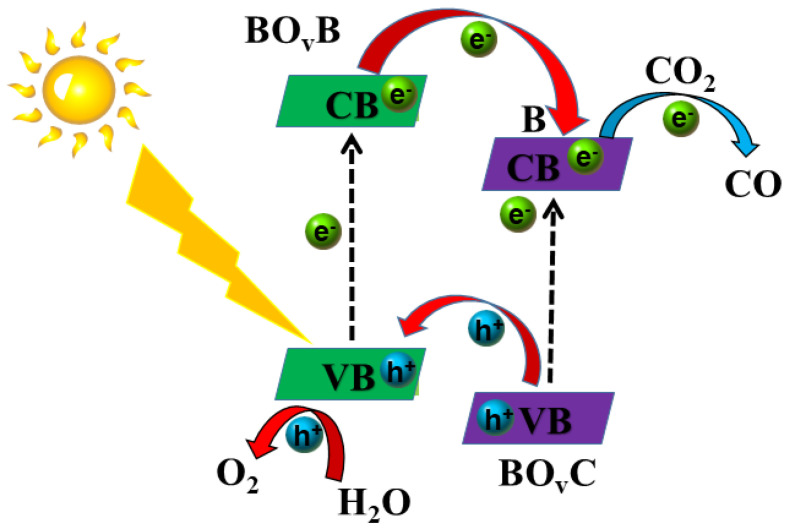
The possible Schematic illustration of photocatalytic CO_2_ reduction into CO for BO_v_B/BO_v_C-5 sample.

Based on the foregoing experimental results, a possible mechanism using the BO_v_B/BO_v_C heterojunction for the photocatalytic CO_2_ reaction is proposed in Figure 8. Under visible light irradiation, BO_v_B is also excited to produce photo-generated electrons (e^−^) and holes (h^+^). The photo-generated electron transfers to the conduction band minimum (CBM), leaving a hole in the valence band maximum (VBM). The left hole could directly oxidize water molecules giving rise to O_2_ and protons. In addition, the photo-generated electrons on CBM flow to the CB of BO_v_C, which leads to the effective separation of photon-generated carriers. The e^−^ on the CB of BO_v_C would reduce CO_2_ into CO. The origin of this enhancement of the photocatalytic CO_2_ reduction rate is the result of the effective separation of electron-hole pairs and the improvement of CO_2_ adsorption capacity derived from the oxygen vacancy.

## 3. Experimental Sections

### 3.1. Materials

KBr, Na_2_SO_4_, and Bi(NO_3_)_3_·5H_2_O were purchased from the Sinopharm Chemical Reagent Corporation (Shanghai, China). All materials were analytical grade and without further purification in the experimental. All used materials are analytical reagents.

### 3.2. Synthesis of BiOBr and Defect-Rich BiOBr Photocatalysts

Pristine BiOBr (BOB) was synthesized through the following steps: 2 mmol KBr was dispersed into 70 mL deionized water, and then, 2 mmol Bi(NO_3_)_3_·5H_2_O was added into the solution and continually stirred for 0.5 h at ambient temperature. Subsequently, the precursor suspension was transferred to a 100 mL autoclave and maintained at 160 °C for 12 h in an oven. The obtained product has been washed with absolute ethanol and deionized water, respectively. At last, the obtained BiOBr sample was dried at 60 °C for 6 h in an oven.

The defect-rich BiOBr was prepared via in situ photo-induced method and 0.3 g BiOBr was dispersed into 100 mL H_2_O. The 300 W Xe arc lamp was used as a light source to irradiate the above solution for 1 h, 3 h, 5 h, and 7 h, respectively, for obtaining the defect BiOBr of different oxygen vacancy content. The solutions of different irradiation periods were filtered and washed several times with deionized water, ultimately dried at 60 °C for 6 h in a vacuum oven; the obtained defect-rich BiOBr samples were marked as BO_v_B-1, BO_v_B-3, BO_v_B-5, and BO_v_B-7.

### 3.3. Characterization

The phase structures of samples were investigated by power X-ray diffraction (XRD) with Cu Kα radiation (λ = 0.154056 nm) on a Bruker AXS D8 advance power diffractometer, and XRD spectra were measured in the range of 2θ = 10–80. The morphologies and composition of the samples were observed by SEM and EDS using a Hitahi S-4800 microscope (Hitachi Limited, Tokyo, Japan) with an accelerating voltage of 7.0 kV. Raman spectra of the samples were recorded on the LABRAM-HR800 system with laser excitation of 532 nm. The spectra were recorded in a shift range of 50–600 cm^−1^. High-resolution transmission electron microscopy (HRTEM) measurements were performed by a JEOL-2100 microscope (Japan Electronics Co., Ltd. (JEOL) Tokyo, Japan) at an acceleration voltage of 200 kV. The preparation process of this test sample is as follows: A small amount of sample was added to 1 mL of ethanol, ultrasonic dispersion for 2 min, and then an appropriate amount of suspension was added to the net copper surface, drying with an infrared lamp. X-ray photoelectron spectroscopy (XPS) was obtained on a Thermo Fisher Scientific, Waltham, MA, USA (ESCALAB 250) spectrometer with the multichannel detector, and C 1s as a signal-calibration standard of binding-energy values at 284.6 eV. Ultraviolet-visible (UV-vis) absorption spectra were recorded from 800–200 nm by a Shimadzu UV-2600 spectrophotometer and using Ba_2_SO_4_ as the reflectance standard sample. The CO_2_ adsorption isotherms were carried out by A Micromeritics ASAP 2020 analyzer (Beijing Builder electronic technology Co., Ltd., Beijing, China). The in situ FT-IR was carried out using FT-IR 4200 Jasco spectrometer (Tokyo, Japan) equipped with a diffuse reflectance accessory. The spectrum was recorded in the wavenumber range of 2200–1000 cm^−1^. Photocurrent and Electrochemical impedance spectroscopy were investigated by CHI660E electrochemical, using 0.5 M Na_2_SO_4_ aqueous solution as an electrolyte solution, Pt as a counter electrode, and Ag/AgCl as reference electrodes. The photocatalysts were deposited on ITO conductive glass to be applied as the working electrode. The preparation method of the working electrode is as follows: a suitable amount of photocatalyst was first mixed with a small amount of ethanol solution. The obtained mixed suspension was ground for 15 min, then a proper amount of supernatant was taken out and spin-coated on ITO glass using the Spin Coater (KW-4A, Institute of Microelectronics, Chinese Academy of Sciences, Beijing, China). At last, the obtained working electrode was dried at 60 °C for 2 h in a vacuum oven. The used light source was a 300 W xenon lamp (PLS-SEX300, Beijing Trusttech CO., Ltd., Beijing, China) (wavelength > 420 nm). A short photocurrent density measurement was performed during the ON/OFF cycle for 110 s.

### 3.4. Photocatalytic CO_2_ Reduction

The photocatalytic CO_2_ test is carried out using a quartz reactor. First, 100 mg of the sample was mixed with 100 mL of deionized water. Subsequently, we sealed it and continuously bubbled high-purity CO_2_ into the reactor for 15 min. During the whole reaction process, the reactor was kept at 15 °C by using cooling water circulation equipment. The used light source was a 300 W Xe arc lamp (PLS-SEX300, Beijing Trusttech Co., Ltd.) (wavelength > 420 nm). At the one-hour interval, the gas samples were obtained using needle tubing. And the reaction products have been analyzed by Varian CP-3800 gas chromatograph (FID detector, Porapak Q column, and the N_2_ gas was used as the carrier gas). The stability of the photocatalyst was also carried out according to the above method.

## 4. Conclusions

Defects chemistry has been proven efficient strategy to provide active sites and accelerate the catalytic activity. For BiOX materials, oxygen vacancy was usually considered to enhance CO_2_ adsorption and widen the optical response range in CO_2_ reduction. Here, taking advantage of the metastable property, the defect-rich BO_v_B/BO_v_C photocatalyst was prepared through the reaction of Bi^3−x^ and ${\mathrm{CO}}_{3}^{2-}$. After the surface reconstruction progress, the photocatalyst was composed of oxygen vacancy-rich BO_v_B and surface amorphous BO_v_C. The formed heterojunction catalyst achieves multiple functions: the oxygen vacancy realizes better visible light absorption of the BiOBr and CO_2_ activation; BO_v_C was generated through an in situ phase changing progress, and this kind of tight contact interface is beneficial for carriers’ migration; the formed BO_v_C layer will provide protection and avoid the oxidization of vacancies by the O_2_. As a result, the defect-rich BO_v_B/BO_v_C shows better activity and good stability in photocatalytic CO_2_ reduction. This study provides a new view for the design of highly efficient photocatalysts which collaborate defect and heterojunction advantages.

## Data Availability

Data will be made available on request.

## Supplementary Materials

The following supporting information can be downloaded at: https://www.mdpi.com/article/10.3390/molecules28104057/s1, Figure S1: SEM images of (a) BOB, (b) BOB-1, (c) BOB-3, (d) BOB-5, (e) BOB-7. Figure S2: CO_2_ absorption properties of samples.
