# Supplementary material for: Light Control-Induced Oxygen Vacancy Generation and In Situ Surface Heterojunction Reconstruction for Boosting CO2 Reduction
Source: Molecules. 2023 May 12;28(10):4057. doi: 10.3390/molecules28104057 (PMC10221696; doi:10.3390/molecules28104057)
Supplement: Supplementary file 1 [file molecules-28-04057-s001.zip › molecules-2388344-supplementary.pdf]

Supplementary Materials

Light Control-Induced Oxygen Vacancy Generation and In Situ Surface Heterojunction Reconstruction for Boosting CO_2 Reduction

Zhimin Yuan [1,†], Xianglin Zhu [2,†], Qichao Gao [3] and Zaiyong Jiang [1,*]

[1] School of Chemistry & Chemical Engineering and Environmental Engineering, Weifang University, Weifang 261061, China
[2] Institute for Energy Research, School of Chemistry and Chemical Engineering, Jiangsu University, Zhenjiang 212013, China
[3] School of Light Industry and Engineering, South China University of Technology, Guangzhou 510640, China
* Correspondence: zaiyongjiang@wfu.edu.cn
† These authors contributed equally to this work.

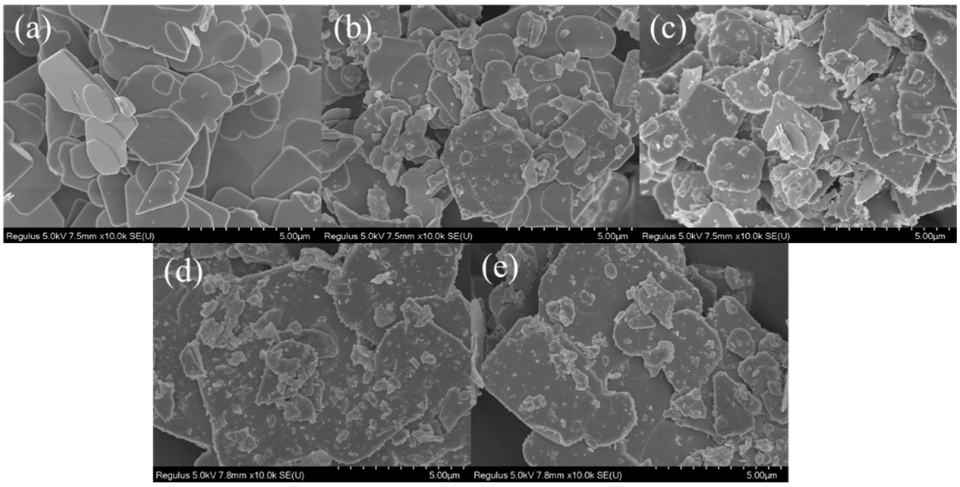

Figure S1. SEM images of (a) BOB, (b) BOB-1, (c) BOB-3, (d) BOB-5, (e) BOB-7.

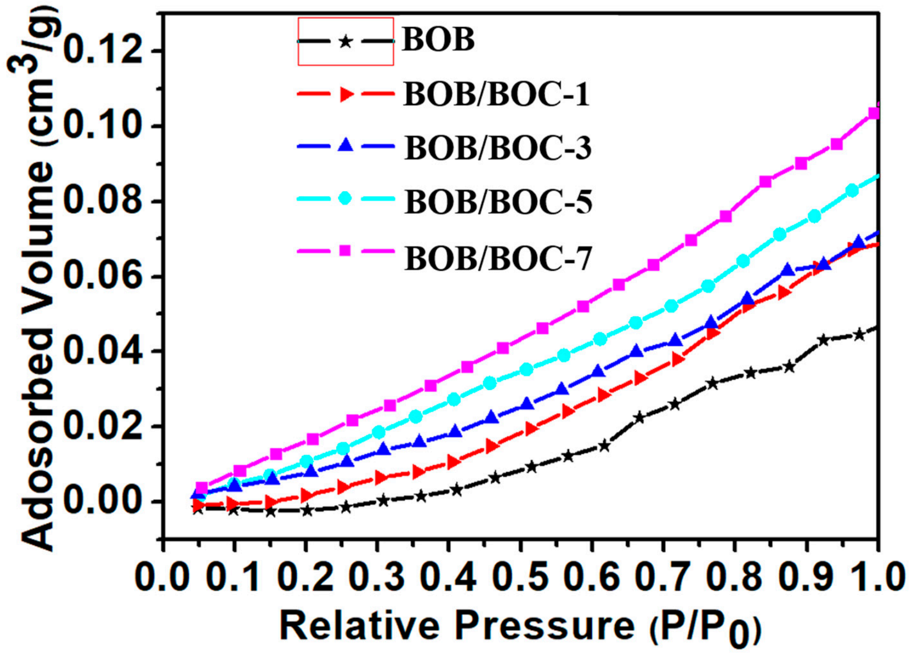

Figure S2. CO_2 absorption properties of samples.
